# Characterization of the Soybean GmIREG Family Genes and the Function of GmIREG3 in Conferring Tolerance to Aluminum Stress

**DOI:** 10.3390/ijms21020497

**Published:** 2020-01-13

**Authors:** Zhandong Cai, Peiqi Xian, Rongbin Lin, Yanbo Cheng, Tengxiang Lian, Qibin Ma, Hai Nian

**Affiliations:** 1 The State Key Laboratory for Conservation and Utilization of Subtropical Agro-Bioresources, South China Agricultural University, Guangzhou 510642, China; zdcai@stu.scau.edu.cn (Z.C.); pqxian@stu.scau.edu.cn (P.X.); linrbl@163.com (R.L.); ybcheng@scau.edu.cn (Y.C.); liantx@scau.edu.cn (T.L.); maqibin@scau.edu.cn (Q.M.); 2 The Key Laboratory of Plant Molecular Breeding of Guangdong Province, College of Agriculture, South China Agricultural University, Guangzhou 510642, China; 3 The Guangdong Subcenter of the National Center for Soybean Improvement, College of Agriculture, South China Agricultural University, Guangzhou 510642, China

**Keywords:** expression pattern, metal stress, transporter, tonoplast, detoxification

## Abstract

The IREG (IRON REGULATED/ferroportin) family of genes plays vital roles in regulating the homeostasis of iron and conferring metal stress. This study aims to identify soybean IREG family genes and characterize the function of *GmIREG3* in conferring tolerance to aluminum stress. Bioinformatics and expression analyses were conducted to identify six soybean IREG family genes. One *GmIREG*, whose expression was significantly enhanced by aluminum stress, *GmIREG3*, was studied in more detail to determine its possible role in conferring tolerance to such stress. In total, six potential IREG-encoding genes with the domain of Ferroportin1 (PF06963) were characterized in the soybean genome. Analysis of the *GmIREG3* root tissue expression patterns, subcellular localizations, and root relative elongation and aluminum content of transgenic *Arabidopsis* overexpressing *GmIREG3* demonstrated that GmIREG3 is a tonoplast localization protein that increases transgenic *Arabidopsis* aluminum resistance but does not alter tolerance to Co and Ni. The systematic analysis of the GmIREG gene family reported herein provides valuable information for further studies on the biological roles of GmIREGs in conferring tolerance to metal stress. *GmIREG3* contributes to aluminum resistance and plays a role similar to that of *FeIREG3*. The functions of other GmIREG genes need to be further clarified in terms of whether they confer tolerance to metal stress or other biological functions.

## 1. Introduction

Plants absorb mineral elements from soil and transport them to various organs and tissues for normal growth and development, which requires different types of transporters, including the natural resistance-associated macrophage protein family of transporters, the zinc/iron-regulated transporter-like protein family, heavy metal ATPase transporters, multidrug and toxic compound extrusion protein transporters, the ATP-binding cassette family of transporters, and others [1,2,3,4]. However, because of the low affinity of certain transporters, toxic metal ions can accumulate in the roots while transporting essential mineral elements for normal growth and development under deficiency conditions [5]. Fortunately, plants have developed elaborate regulatory mechanisms to perceive the metal stress signals and adjust metal detox pathways by modulating the expression of genes, such as *NADH dehydrogenase subunit 1* [6], plasma membrane H^+^-ATPase [7], half-size ABC transporters [8,9], and IRON REGULATED/ferroportin (IREG) transporters [2,5,10,11].

The IREG family, with its highly conserved domain, exists in a wide range of bacteria, animals, and plants [5]. Some members of this family in plants have been functionally characterized, especially in model plants such as *Arabidopsis* (*Arabidopsis thaliana*). There are only three members of the IREG transporter protein family in *Arabidopsis*. AtIREG1 is localized in the plasma membrane and is expressed in the root stele, which is essential for cobalt tolerance and iron homeostasis [10]. Unlike AtIREG1, AtIREG2 is localized in the vacuolar membrane of root cells and functions as a transporter for cobalt and nickel into the vacuole [5]. AtIREG3 (also known as MAR1) is a plastid transporter that might play roles in the regulation of cellular iron homeostasis, and may be involved in controlling the opportunistic entry of multiple antibiotics into the chloroplast [12,13]. In *Psychotria gabriellae*, PgIREG1 behaves as a functional orthologue of the AtIREG2 at the cellular level, and is also localized in the vacuolar membrane and involved in Ni tolerance [2]. Recently, FeIREG1 from *Fagopyrum esculentum* Moench was shown to be an Al-specific IREG transporter involved in tolerance to Al toxicity but not to other metals [11]. These findings indicate that the functions of IREGs are different among plant species.

Aluminum is the most widely distributed metal element on earth. Because of its excellent properties, it is among the most universal engineering and construction materials in industry. Unlike essential transition metals, Al is a nonessential element for plants, and micromolar concentrations of Al can harm the root growth of plants [8]. With the increasing acidification of soils and waters, Al toxicity accompanied by manganese toxicity or phosphorus deficiency has become a major limitation to crop growth in acidic soils [14,15]. Two main types of Al resistance mechanisms have been established in plant species during long-term evolution, i.e., exclusion mechanisms and internal tolerance mechanisms [16,17,18]. Multidrug and toxic compound extrusion (MATE) transporters and aluminum-activated malate transporter (ALMT) are crucial for Al-induced organic acid efflux, which can detoxify Al internally and externally [15,19,20]. Vacuoles play an important role in internal tolerance mechanisms due to their function of storing Al in plant cells. Proteins localized on the vacuole membrane such as AtALS1, OsALS1, and FeIREG1 play an active role in resistance to Al toxicity [8,9,11].

Soybean (*Glycine max* L.) is one of the most important sources of protein, oil, and micronutrients, and is particularly sensitive to Al toxicity. Although a few Al-tolerant genes involved in exclusion mechanisms and internal tolerance mechanisms have been functionally characterized, gene resources involved in Al tolerance in soybean are not sufficient compared with those in rice and wheat. Furthermore, few data are available concerning the IREG gene family in soybean to date. In the present study, a bioinformatics analysis was conducted to identify six soybean IREG genes. GmIREG3, whose expression was significantly enhanced by aluminum stress, was studied in transgenic *Arabidopsis* to determine its possible role in conferring tolerance to aluminum stress.

## 2. Results

### 2.1. Bioinformatics Analysis of Six IREG Genes in Soybean

Six potentially encoding IREG proteins were blasted from the soybean genome in the Phytozome database using the protein domain of Ferroportin1 (PF06963) and termed GmIREG1 to GmIREG6. Chromosome mapping showed that the six GmIREGs are distributed on four chromosomes. Chromosomes 1 and 3 each contained two GmIREGs, while chromosomes 10 and 20 each contained one GmIREG (Table 1 and Figure S1). The CDS regions of six soybean IREG genes ranged from 528 (*GmIREG4*) to 1764 (*GmIREG5*) bp in length, encoding proteins with lengths of 175 (GmIREG4) to 587 (GmIREG5) aa. The molecular weights (MWs) ranged from 19.06 kDa to 63.19 kDa, and PI values ranged from 6.16–9.07.

**Figure 1:**
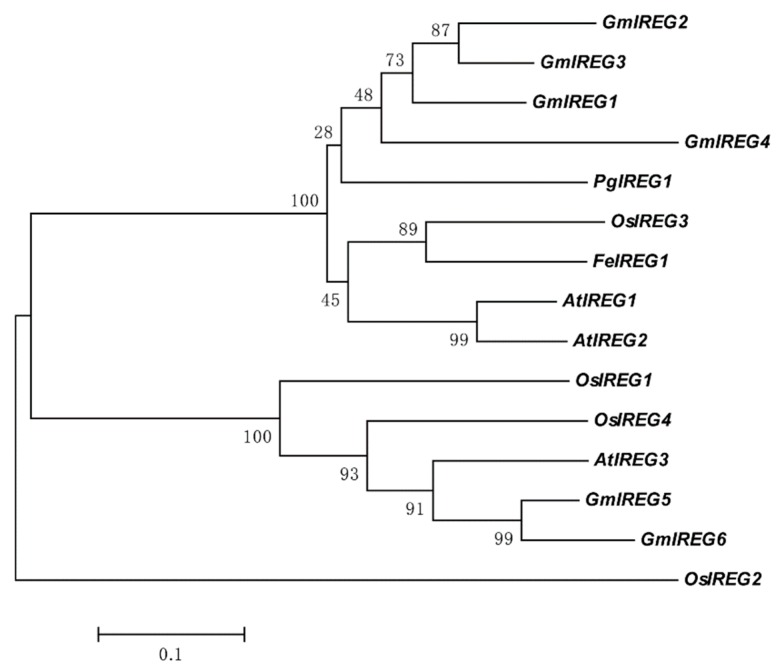
Phylogenetic analysis of the GmIREGs and published IREG proteins. The phylogenetic tree was constructed using the neighbor-joining method with 1000 bootstrap replicates by MEGA 7.0 software. The accession numbers for soybean IREG genes can be found in Table 1, and the other accession numbers are as follows: AtIREG1 (At2g38460), AtIREG2 (At5g03570), AtIREG3 (At5g26820), OsIREG1 (LOC_Os05g04120), OsIREG2 (LOC_Os06g14170), OsIREG3 (LOC_Os06g36450), OsIREG4 (LOC_Os12g37530), and PgIREG1 (CCM80483.1). The amino acid sequence of FeIREG1 can be found in [11].

The multiple sequence alignment and phylogenetic investigation were conducted with the inclusion of IREGs from *Arabidopsis thaliana*, rice, and two published IREGs (*PgIREG1* and *FeIREG1*). As shown in Figure 1, fifteen IREGs, including six GmIREGs, were classified into three groups. Group I was composed of nine IREG proteins including four GmIREGs (GmIREG1, GmIREG2, GmIREG3, and GmIREG4), and group II was composed of six IREG proteins including GmIREG5 and GmIREG6. Phylogenetic analysis indicated that GmIREG1, GmIREG2, GmIREG3, and GmIREG4 cluster closely with three published IREGs having the function of transporting metal ions: AtIREG2 from *Arabidopsis thaliana*, PgIREG1 from *Psychotria gabriellae,* and FeIREG1 from *Fagopyrum esculentum* Moench.

### 2.2. Expression Patterns of GmIREG Genes

**Figure 2:**
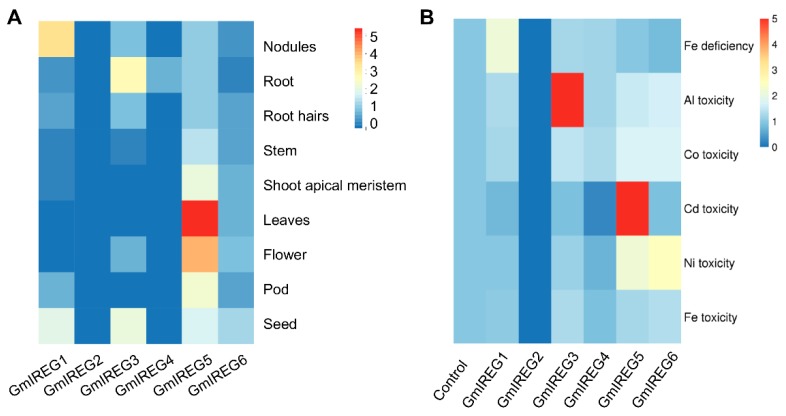
Tissue-specific expression and metal-induced expression patterns of six soybean IREG genes. (**A**) Heat map analysis shows the different tissue specific expression pattern of GmIREGs using the PKFM data of phytozome v12 database. (**B**) Expression of GmIREGs in response to Fe deficiency, Al toxicity, Co toxicity, Cd toxicity, Ni toxicity, and Fe toxicity. For Fe deficiency, uniform seedlings were exposed to normal conditions (half-strength nutrient solution, pH 5.6) or low-iron conditions (half-strength nutrient solution without Fe-Na-EDTA, pH 5.6) for 14 days. For other toxicity treatments, the uniform seedlings were treated with excess Fe (1000 µM Fe-EDTA), Ni (30 µM NiCl_2_·6H_2_O), Cd (30 µM CdCl_2_), Co (30 µM CoCl_2_), and Al (30 µM AlCl_3_·6H_2_O) treatments for 12 h. The roots were separately harvested for qRT-PCR analysis. The heat map displays 2^ΔΔ*C*t^ values to show relative expression of metal treated samples vs. controls.

Based on the expression data in Phytozome, a heat map of the tissue expressions was analyzed (Figure 2A). All GmIREGs were detected in each tissue except *GmIREG2*. Expression was high for *GmIREG5* in all tissues, and was highest for *GmIREG5* in flowers. Relatively high expression was also observed for *GmIREG1* in nodules and *GmIREG3* in root. Moreover, we tested whether the soybean GmIREGs expression were induced by metals using qRT-PCR (Figure 2B). Exposure of soybean roots to Al only induced the expression of *GmIREG3*. With exposure to Cd, the expression of *GmIREG4* was significantly downregulated, but the *GmIREG5* was significantly upregulated. *GmIREG5* and *GmIREG6* were upregulated by Ni. However, the expression levels of six soybean GmIREGs were not altered by Co and Fe.

### 2.3. Cloning and Characterization of GmIREG3

**Figure 3:**
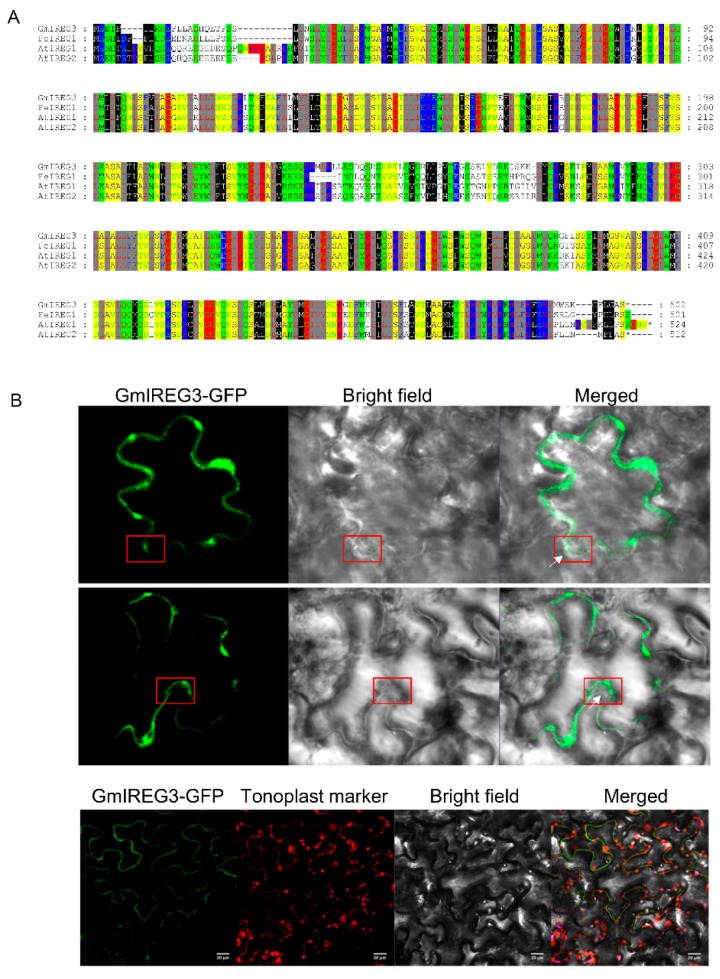
Alignment of amino acid sequence and subcellular localization of GmIREG3. (**A**) Amino acid sequence alignment of IREG proteins from soybean (GmIREG3), buckwheat (FeIREG1) and *Arabidopsis* (AtIREG1 and AtIREG2). (**B**) Subcellular localization of a C-terminal GmIREG3-GFP fusion protein in *Nicotiana benthamiana* and the fusion protein was driven by 35S promoter. At 3 d after infiltration, the fluorescence signals were visualized by confocal microscopy. GmIREG3-GFP was colocated with the tonoplast marker to the tonoplast. Red rectangles indicate the tonoplast separated from the plasma membrane and white arrows indicate the plasma membrane.

On the basis of sequence information of *GmIREG3* from the Phytozome database, we obtained the full-length *GmIREG3* cDNA (GenBank accession number: MN635747) and approximately 2-Kb promoter sequence (GenBank accession number: MN781668) from HX3 (Al-tolerant genotype). In agreement with the prediction of the Phytozome database, the GmIREG3 coding region was 1509 bp in length and encoded a protein of 502 amino acids. A BLAST search of orthologues in *Arabidopsis* showed that GmIREG3 shared 71.5%, 73.5%, and 75.7% similarity with AtIREG1, AtIREG2, and FeIREG1, respectively (Figure 3A). To further reveal the divergence of the GmIREG3 protein during evolution, a BLAST analysis in NCBI with the protein sequence of GmIREG3 was performed; we found that GmIREG3 and genes of leguminous plants such as *Arachis hypogaea* and *Medicago truncatula* are in the same clade (Figure S2). Furthermore, the orthologues are present in both monocots and dicots, and the proteins may be classified into two distinct clades, which suggested the evolution of GmIREG3 and orthologues before the divergence of monocots and dicots. To identify the subcellular location of GmIREG3, its coding region was fused upstream of the GFP gene and expressed under the CaMV35S promoter in leaves of *Nicotiana benthamiana*. Confocal images demonstrated that GmIREG3 was colocated with the tonoplast marker to the tonoplast (Figure 3B).

The 1958-bp promoter of *GmIREG3* was cloned and sequenced. Based on the PlantCARE analysis, the promoter of *GmIREG3* contained a few cis-elements involved in plant hormone signaling, including elements associated with abscisic acid-responsive element (ABRE), salicylic acid-responsive element (TCA), and the gibberellin-responsive element (P-box), which play significant roles in regulating the growth of plant and stress responses. Some cis-elements associated with light responses were also identified in the *GmIREG3* promoter such as ACE, Box-4, and G-box. Additionally, both basal elements, i.e., CAAT-box and TATA-box, and stress-related cis-acting elements W-box, MBS, and LTR are contained in the in the *GmIREG3* promoter (Table 2).

### 2.4. Root GmIREG3 Expression Levels Are Increased by Al

**Figure 4:**
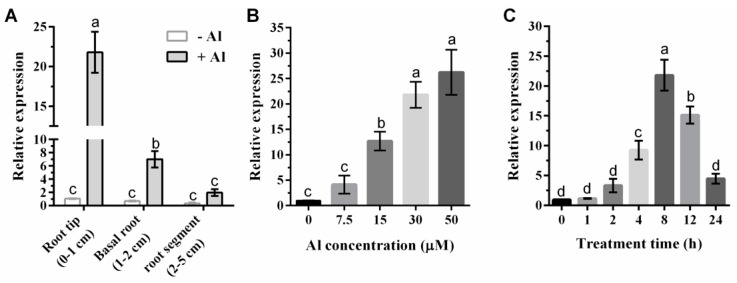
Soybean *GmIREG3* expression analysis. (**A**) Tissue-specific expression of *GmIREG3* in response to Al stress. Samples for spatial expression were taken from different root segments (0–1, 1–2 and 2–5 cm from the apex) of seedlings with or without 30 µM Al treatment. (**B**) Dose-dependent *GmIREG3* expression in soybean root tips (0–1 cm). The roots were exposed to different Al concentrations (0, 7.5, 15, 30, and 50 µM) for 8 h. (**C**) For the time-course experiment, seedlings were exposed to 30 µM AlCl_3_ for 0, 1, 2, 4, 8, 12, or 24 h. The samples were separately harvested for qRT-PCR analysis. Values are expressed as the means ± SD (*n* = 3). Different letters indicate a statistically significant difference, using one-way ANOVA and Duncan’s test (*p* < 0.05).

To further examine the expression patterns of *GmIREG3* following Al stress, three-day-old root tips of soybean were exposed to different concentrations and durations of Al. Spatial expression analysis showed that the expression levels of *GmIREG3* were induced in 0–5 cm root segments by 30 µM Al (Figure 4A). However, the expression induction was much greater in the root tips than in other root segments (Figure 4A). In a dose-response experiment, as shown in Figure 4B, the expression of *GmIREG3* increased with increasing Al concentration. Furthermore, the Al-induced expression of *GmIREG3* was found to increase rapidly within 8 h of exposure to 30 µM Al and decrease after 8 h of exposure (Figure 4C).

### 2.5. Overexpression of GmIREG3 in Transgenic Arabidopsis Thaliana Confers Al Tolerance

**Figure 5:**
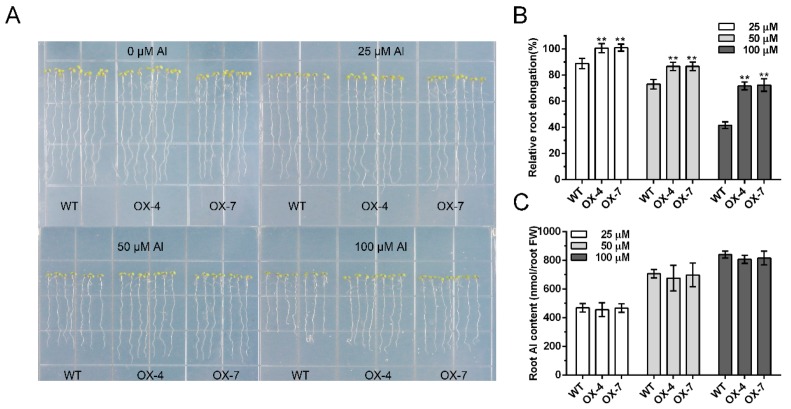
Overexpression of *GmIREG3* enhances Al tolerance in *Arabidopsis*. (**A**) Evaluation of Al tolerance in *GmIREG3* overexpression lines. After germination in MS medium, five-day-old seedlings were transferred to solid agar medium supplied with 1 mM CaCl_2_ and 1% sucrose containing different concentrations of AlCl_3_ (0, 25, 50 or 100 μM; pH 4.5) for 3 d. The photograph was taken for different treatments at the end of the experiment. The relative root elongation (*n* = 14) (**B**) and root Al concentration were measured (*n* = 3) (**C**). All data are presented as means ± SD. Significant differences according to the one-way analysis of variance are denoted as follows: ** *p* < 0.01.

**Figure 6:**
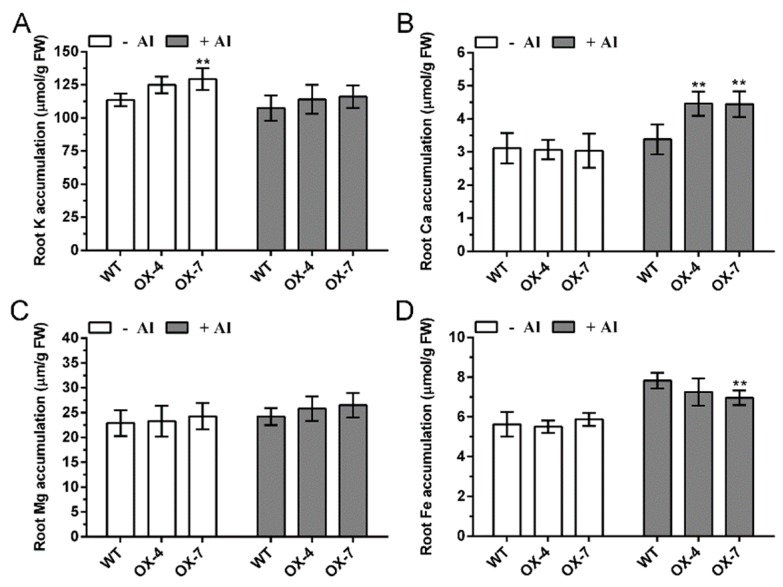
Concentration of other metals in transgenic *Arabidopsis* overexpressing *GmIREG3*. *Arabidopsis* plants were exposed to 0 or 10 µM Al solution for 2 d and then subjected to determination of K (**A**), Ca (**B**), Mg (**C**) and Fe (**D**). Data are means ± SD (*n* = 3). Significant differences according to the one-way analysis of variance are denoted as follows: ** *p* < 0.01.

To evaluate the role of *GmIREG3* in the Al stress response, a 35::GmIREG3 construct was introduced into *Arabidopsis thaliana* by the *Agrobacterium tumefaciens*-mediated floral-dip method [21,22]. Two independent homozygous T4 transgenic lines with higher expression (OX-4 and OX-7) were selected for phenotypic and physiological analysis (Figure S3). To test the Al tolerance wild-type (WT) and transgenic lines, the uniform seedlings (approximately 1.0 cm) of these lines were transferred to CaCl_2_ agar plates with 0, 25, 50, or 100 µM AlCl_3_. As shown in Figure 5, we did not observe any difference in root growth between WT and transgenic lines in the absence of Al. However, in the presence of 25 µM Al, the root elongation of WT was inhibited by 12% after three days, but transgenic lines were not significantly influenced. With the increase of Al concentration, the root elongation of both WT and transgenic lines was inhibited. At 100 µM Al, the root elongation of WT was inhibited by 58% and that of the transgenic lines was inhibited by 19% (Figure 5B). The concentration of Al in the fresh weight of the roots was also observed. In the case of equal biomass, overexpression of *GmIREG3* in *Arabidopsis thaliana* slightly decreased the Al accumulation in roots compared with the WT at relatively high concentrations of Al (100 µM Al), but no difference was observed at low concentrations of Al (Figure 5C). No large difference was observed in the accumulation of K, Mg, and Fe in the roots, but there was a significant difference in Ca content between the WT and *GmIREG3*-overexpressing lines (Figure 6).

## 3. Discussion

IREG proteins are widespread in all organisms including bacteria, animals, and plants. The IREG gene family has been reported in *Arabidopsis thaliana*, *Psychotria gabriellae,* and *Fagopyrum esculentum* Moench, but to the best of our knowledge, the information on this family is limited for soybean. The present study aimed to perform the genome-wide analysis of soybean IREG genes and investigate the function of *GmIREG3* gene response to Al toxicity. Our results show that six IREG genes in the soybean genome harbored the typical Ferroportin1 (PF06963) domain (Table 1 and Figure S1). Phylogenetic analysis clustered these six IREG proteins into two distinct subfamilies (Figure 1). The five IREG genes were constitutively expressed in soybean plants, while the expression data of *GmIREG2* could not be detected in all our samples (Figure 2). Although IREG genes function in both iron absorption in the intestine and iron recycling in macrophages in mammals, several previous studies revealed that IREG genes play different roles in plants [11,23]. In soybean, *GmIREG1* was significantly affected by Fe deficiency in roots and shoots (Figure 2B), indicating that GmIREG1 might also be involved in Fe deficiency. Recent studies have revealed important roles for IREG genes in regulating metal homeostasis in plant cells, especially Ni, Co, and Al [2,5,10,11]. The transcriptional expression of two soybean IREG genes (*GmIREG5* and *GmIREG6*) was induced by Ni (Figure 2B), combining with their different levels of tissue expression (Figure 2A), implying that *GmIREG5* might be involved in Ni tolerance or accumulation above ground, while *GmIREG6* might be involved in the roots. One soybean IREG gene (*GmIREG3*) was remarkably upregulated by Al in roots (Figure 2B), indicating that *GmIREG3* might confer tolerance to Al stress. Beyond functioning in Ni, Co, and Al tolerance or accumulation, two IREG genes (*GmIREG4* and *GmIREG5*) were induced by Cd in roots (Figure 2B), indicating that soybean IREG genes might have certain novel functions.

The *GmIREG3* was remarkably upregulated by Al in roots (Figure 4), and was selected for further characterization. A BLAST search of orthologues in plants showed that GmIREG3 shared 71.5%, 73.5%, and 75.7% similarity with AtIREG1, AtIREG2, and FeIREG1, respectively (Figure 3A). Subcellular localization analysis showed that GmIREG3 protein was localized in the tonoplast, as was the case for PgIREG1 (Figure 3B). Recently, FeIREG1 was functionally characterized as an Al transporter involved in Al tolerance [10]. These results suggested that GmIREG3 might function as Al transporter involved in Al tolerance. In addition, two classical abiotic stress cis-acting elements (W-box and MBS) were found in the promoter of *GmIREG3* (Table 2). The WRKY gene family members play critical roles in certain plant processes in response to abiotic stress by directly binding to W-box (TGACC (A/T)) in the promoter of its target genes. In *Arabidopsis*, AtWRKY46 as a negative regulator can inhibit *AtALMT1* expression, which leads to decreased malate secretion and increased Al accumulation in root tips [24]. In contrast, *OsWRKY22* can increase the expression of *OsFRDL4* by directly binding to its promoter, which enhances citrate secretion and Al tolerance in rice [25]. Although MYB transcription factors are not directly reported to be associated with Al resistance, Hu et al. [26] found that MdMYB73 protein bound directly to the promoters of *MdALMT9*, which can influence malate accumulation and vacuolar pH. Thus, we hypothesize that GmIREG3 might play an important role in Al tolerance.

To further confirm GmIREG3 function in Al tolerance, transgenic *Arabidopsis* overexpressing GmIREG3 were generated. Consistent with this speculation, the overexpression of *GmIREG3* enhanced Al tolerance in *Arabidopsis* (Figure 5). A recent study suggested that *FeIREG1* from *Fagopyrum esculentum* Moench, an Al-specific tolerance IREG gene, showed similar patterns in transgenic *Arabidopsis* to those of *GmIREG3*, but also exhibited differences in the accumulation of mineral elements under Al tolerance with *GmIREG3*, such as Ca and Fe (Figure 6) [11]. In *Arabidopsis*, *AtIREG1* is involved in the xylem loading of Fe and Co, and *AtIREG2* is involved in the sequestration of Co and Ni into the vacuoles [5,10,11]. It is important to note that neither *AtIREG1* nor *AtIREG2* is involved in Al tolerance in *Arabidopsis* [11]. Although *GmIREG3* is the *Arabidopsis* orthologue of *AtIREG1* and *AtIREG2* (Figure 1andFigure 3A), unlike *AtIREG1* and *AtIREG2*, our results showed that *GmIREG3* is involved in the tolerance to Al toxicity but not to Ni and Co toxicity (Figure 5 and Figure S4). Moreover, *GmIREG3* is expressed in the both root tip and stele (Figure 4A), and its expression was upregulated in 0–5 cm roots (Figure 4B), implying that *GmIREG3* might participate in the sequestration of Al to the vacuoles in the root tip. These findings indicate that the functions of IREGs differ among plant species, as noted in a previous study [11]. More in-depth research on the IREG family is needed.

It is well known that certain highly tolerant species can accumulate high concentrations of Al in their tissues without showing symptoms of Al toxicity, such as buckwheat, *Melastoma malabathricum,* and *Hydrangea* plants [18]. Most plants absorb Al^3+^ mainly from the root tips, leading to a large accumulation of Al there [27,28,29]. Therefore, there must be an internal detoxification mechanism in the root tips and other tissues. Vacuoles play an important role in the storage of toxic substances in plant cells. Metal sequestration in the vacuole has been well established to detoxify toxic metal internally, which is a relatively mature toxic metal resistance mechanism in higher plants [30,31,32,33,34]. In previous studies, two half-size ABC transporters, AtALS1 and OsALS1, were reported to be involved in Al tolerance by sequestration of Al to the vacuoles in the root cells [8,9]. In the present study, like FeIREG1, AtALS1, and OsALS1, GmIREG3 was shown to be localized in the tonoplast (Figure 3B). Furthermore, transgenic *Arabidopsis* overexpressing *GmIREG3* showed an increased tolerance to elevated concentrations of Al (Figure 5). The most likely cause of this phenotype is that *GmIREG3* is involved in Al tolerance by sequestrating Al into the vacuoles of roots.

Soybean (*Glycine max* L.) is one of the most important sources of protein, oil, and micronutrients, and is particularly sensitive to Al toxicity. To date, some Al-tolerant genes have been reported in soybean, but information on the IREG family is limited. This study systematically characterized the GmIREGs and their metal-induced expression patterns in soybean. Moreover, we established that *GmIREG3* plays a role similar to that of *FeIREG1* in contributing to Al tolerance. These results supply basic and important information for understanding the putative functions of IREG genes in soybean.

## 4. Materials and Methods

### 4.1. Identification and Bioinformatics Analyses of IREG Genes in Soybean

To identify IREG homologues in soybean, the nucleic acid sequences of reported IREGs in *Arabidopsis* (*AtIREG1*, *AtIREG2,* and *AtIREG3*), buckwheat (*FeIREG1*), and *Psychotria gabriellae* (*PgIREG1*) were used as query sequences to BLAST from soybean genomes in Phytozome v12 (available online: https://phytozome.jgi.doe.gov/pz/portal.html#!search?show=BLAST&method=Org_Gmax, accessed on 21 October 2016). Six proteins with IREG domains were identified through filtering with the presence of conserved IREG domain (Pfam: PF06963) using Pfam (available online: http://pfam.xfam.org/, accessed on 21 October 2016) and the Simple Modular Architecture Research Tool (SMART, available online: http://smart.embl-heidelberg.de/smart/batch.pl, accessed on 21 October 2016) [35,36]. *GmIREG1* (*Glyma.01G128300*), *GmIREG2* (*Glyma.01G128400*), *GmIREG3* (*Glyma.03G042400*), *GmIREG4* (*Glyma.03G042500*), *GmIREG5* (*Glyma.10G146600*), and *GmIREG6* (*Glyma.20G096400*) were named accordingly. The chromosomal locations of GmIREG genes were illustrated by MapChart [37]. The deduced protein sequences of all six GmIREGs and reported IREGs were used for multiple sequence alignments through ClustalX and GeneDoc software. The phylogenetic tree with other IREG genes was analyzed using the neighbor–joining method in the MEGA7.0 program with 1000 bootstrap replicates. PlantCARE (available online: http://bioinformatics.psb.ugent.be/webtools/plantcare/html/, accessed on 28 December 2017) was used for cis-element analysis in the 1958 bp region upstream of the start codon for *GmIREG3*.

### 4.2. Plant Materials and Stress Treatments

Soybean cv. HX3 (Al-tolerant genotype) was employed in this study. Soybean seeds were surface sterilized in 75% alcohol followed by germination for three days in sterile vermiculite. Seedlings of similar size were transplanted into the corresponding nutrient solution for gene cloning and transcriptional expression experiments. To investigate possible responses of GmIREGs to Fe deficiency, uniform seedlings were exposed to normal conditions (half-strength nutrient solution, pH 5.6) or low-iron conditions (half-strength nutrient solution without Fe-Na-EDTA, pH 5.6) for 14 days, during which time nutrient deficiency was observed [38]. For other treatments, uniform seedlings were exposed to a nutrient solution as previously described [39]; the nutrient solution contained 200 mM CaSO_4_, 200 mM CaCl_2_, 100 mM MgSO_4_, 400 mM KNO_3_, 300 mM NH_4_NO_3_, 5 mM NaH_2_PO_4_, 3 mM H_3_BO_3_, 0.5 mM MnCl_2_, 0.4 mM ZnSO_4_, 0.2 mM CuSO_4_, 10 mM Fe-EDTA, and 1 mM (NH_4_)_6_Mo_7_O_24_. The solution was adjusted to pH 4.5 with HCl and renewed daily. After 2 d of culture, the uniform seedlings were subjected to the following treatments. To elucidate the probable functions of GmIREGs in response to metal toxicity stresses, seedlings were treated with excess Fe (1000 µM Fe-EDTA), Ni (30 µM NiCl_2_·6H_2_O), Cd (30 µM CdCl_2_), Co (30 µM CoCl_2_), and Al (30 µM AlCl_3_·6H_2_O) for 12 h. For the time-course experiment, seedlings were exposed to 30 µM AlCl_3_ for 0, 1, 2, 4, 8, 12, or 24 h. A dose-dependent experiment was performed by exposing the seedlings to different Al concentrations (0, 7.5, 15, 30, and 50 µM). Samples for spatial expression were taken from different root segments (0–1, 1–2, and 2–5 cm from the apex) of the seedlings. Each treatment had four biological replicates.

### 4.3. Analysis of Expression

Total RNA was isolated from the samples using the Plant Total RNA Purification Kit (TR02-150, GeneMarkbio, Taiwan). One microgram of total RNA was synthesized into first-strand cDNA using the PrimeScript™ RT reagent Kit with a gDNA Eraser kit (RR047, TAKARA, Shiga, Japan). Quantitative real-time PCR (qRT-PCR) was performed to determine the transcriptional expression level of GmIREG genes using the TB Green™ Premix Ex Taq™ II (RR820, TAKARA, Shiga, Japan) in a CFX96 Real-Time System (Bio-Rad, Hercules, CA, USA) with *GmActin6* (GenBank: AK285830.1) or *AtActin2* (*At3g18780*) as an internal control. The primers used in the qRT-PCR reactions are listed in Supplemental Table S1. For all experiments, qRT-PCRs were performed in triplicate on three different RNA samples isolated independently from each tested condition. The relative expression level of the genes was computed by the 2−^ΔΔCt^ method [40]. The FPKM of six *GmIREG*s in nodules, root, root hairs, stem, shoot apical meristem, leaves, flower, pod, and seed were downloaded from phytozome v12 (available online: https://phytozome.jgi.doe.gov/pz/portal.html#!search?show=BLAST&method=Org_Gmax, accessed on 21 October 2016).

### 4.4. Subcellular Localization of GmIREG3

A 1958 bp promoter region of *GmIREG3* was obtained from the genomic DNA of HX3 using Clone-GmIREG3F/R primers. The amplified fragments were subsequently cloned into the pLB vector (VT205, TIANGEN) for sequence confirmation. To construct the GmIREG3-GFP fusion-protein-expressing constructs, *GmIREG3* cDNA was amplified from the cDNA using specific primers (Supplemental Table S1) and inserted into the *Nco I* and *Spe I* sites of the pCambia 1302 vector using the ClonExpress^®^ II One Step Cloning Kit (C112, Vazyme, Nanjing, China). Subcellular localization was investigated by overexpressing *35S:GmIREG3-GFP* transiently in tobacco (*Nicotiana tabacum*) leaves by *Agrobacterium*-mediated transformation [41]. The GFP fluorescence was observed using confocal scanning microscopy (LSM780, Zeiss, Jena, Germany).

### 4.5. Functional Characterization of GmIREG3 in Transgenic Arabidopsis

To construct the *GmIREG3* overexpression (OX) vectors, the open reading frame of *GmIREG3*, which was prepared for subcellular localization investigation as described above, was ligated between CaMV 35S and the NOS terminator of the pTF101.1-GFP vector using the ClonExpress^®^ II One Step Cloning Kit (Vazyme, Nanjing, China). The resulting *GmIREG3* overexpression construct was transformed into *Agrobacterium* strain GV3101, which was then used for *Arabidopsis* transformation according to the *Agrobacterium tumefaciens*-mediated floral dip method [21,22]. The expression of *GmIREG3* in transgenic plants was quantified by qRT-PCR. Two representative transgenic homozygous T3 lines were used for an Al sensitivity assays by measuring relative root elongation according to Yokosho et al. [11]. After germination in MS medium, five-day-old seedlings were transferred to solid agar medium supplied with 1 mM CaCl_2_ and 1% sucrose containing different concentrations of AlCl_3_ (0, 25, 50 or 100 μM; pH 4.5), NiCl_2_ (0, 5 or 10 μM; pH 5.0), and CoCl_2_ (0, 5 or 10 μM; pH 5.0). Their root lengths were measured before and after 3 d of different treatments. The relative root elongation (RRE) was computed as (root elongation with different treatments/root elongation without treatments) ×100. The root samples were used for the determination of Al contents. For the determination of the K, Ca, Mg, and Fe concentration in the transgenic roots, one-week-old plants were exposed to 1/30 Hoagland solution without NH_4_H_2_PO_4_, but with 1 mM CaCl_2_ in the absence or presence of 10 μM AlCl_3_ at pH 4.5. After one week, the roots were harvested separately and then used for the determination of K, Ca, Mg, and Fe contents by ICP-AES.

### 4.6. Statistical Analysis

All data were analyzed using GraphPad Prism^®^ 5 (Version 5.01, GraphPad Software, Inc., USA) for calculating mean and standard deviation. At least three biological replicates were included in the data, and all data were analyzed using ANOVA or Duncan’s test for the determination of the significant differences with SPSS 21 (IBM Corp, 2012). The Heatmap_V2.16 (available online: http://www.lc-bio.cn/overview/8?tools=Heatmap_V2.16, accessed on 10 August 2019) was used to construct the heat map to analyze the tissue-specific expression and the metal-induced expression of GmIREGs.

## Figures and Tables

**Table 1:** Summary of IREG family genes in soybean.

Gene Name	Chromosome	Gene Locus	Length of CDS (bp)	No. of Amino Acids (aa)	MW (kD)	PI	Protein Domain Family
*GmIREG1*	1	*Glyma.01G128300*	1506	501	56.21	7.04	Ferroportin1 (PF06963)
*GmIREG2*	1	*Glyma.01G128400*	1023	340	38.22	8.64	Ferroportin1 (PF06963)
*GmIREG3*	3	*Glyma.03G042400*	1509	502	56.14	7.58	Ferroportin1 (PF06963)
*GmIREG4*	3	*Glyma.03G042500*	528	175	19.06	9.07	Ferroportin1 (PF06963)
*GmIREG5*	10	*Glyma.10G146600*	1764	587	63.19	7.60	Ferroportin1 (PF06963)
*GmIREG6*	20	*Glyma.20G096400*	1251	416	45.19	6.16	Ferroportin1 (PF06963)

**Table 2:** The cis-acting elements identified in *GmIREG3* promoter.

Site name	Species	Position	Sequence	Function
ABRE	*Arabidopsis thaliana*	1643	ACGTG	cis-acting element involved in the abscisic acid responsiveness
ACE	*Petroselinum crispum*	−809	GACACGTATG	cis-acting element involved in light responsiveness
Box 4	*Petroselinum crispum*	−1019/−1918/−1477/−1463/−1515	ATTAAT	part of a conserved DNA module involved in light responsiveness
G-box	*Zea mays*	−1642	CACGTC	cis-acting regulatory element involved in light responsiveness
LTR	*Hordeum vulgare*	−1794	CCGAAA	cis-acting element involved in low-temperature responsiveness
MBS	*Arabidopsis thaliana*	+290	CAACTG	MYB binding site involved in drought-inducibility
P-box	*Oryza sativa*	−1927	CCTTTTG	gibberellin-responsive element
TCA	*Nicotiana tabacum*	−833	CCATCTTTTT	cis-acting element involved in salicylic acid responsiveness
W box	*Arabidopsis thaliana*	−1924	TTGACC	cis-acting element involved in stress responsiveness

## Supplementary Materials

Supplementary materials can be found at https://www.mdpi.com/1422-0067/21/2/497/s1.

## Abbreviations

IREG	IRON REGULATED
FNP	Ferroportin
GFP	Green fluorescence protein
qRT-PCR	Real-time quantitative polymerase chain reaction
ORF	Open reading frame
WT	Wild type
ICP-AES	Inductively coupled plasma-atomic emission spectrometry
FPKM	Fragments Per Kilobase per Million
