# Supplementary material for: Characterization of the Soybean GmIREG Family Genes and the Function of GmIREG3 in Conferring Tolerance to Aluminum Stress
Source: Int J Mol Sci. 2020 Jan 13;21(2):497. doi: 10.3390/ijms21020497 (PMC7013977; doi:10.3390/ijms21020497)
Supplement: Supplementary file 1 [file ijms-21-00497-s001.pdf]

Supplementary Material

1 Supplementary Data

1.1 CDS sequencing information of *GmIREG3*

ATGGAAGAAACCTTCATATTGAGGAGAGAGCCTCTTCTTGCCCAACATCAAGAAACCCC
ATCTTCTTTAATCAATCATTTGTATATTGGTCACTTCTTAGCTAGATGGGGTGCCAGGAT
GTGGGAATTCTCTGTTGGTTTATATATGATCAACATTTGGCCAGACTCATTGCTGTATGC
AGCAATATATGGTGCTGTAGAATCTGCCTCCATTGCACTGTTTGGTTCCATAATTGGAAG
ATGGGTTGACAAGTTGTGTTATGTGAAGGTTTTGCAGTTGTGGTTGGTGACACAGAATCT
CTCTTTTGTTGTTGCTGGGGCCACTGTAGTTGCTTTACTACTCAACTCATCTTTGAAGTTG
ACAAATTTCTCAGCTTTCATATTGATGGTGTTGACAATCAATATCTGTGGTGGCATTGGT
GTACTTTCAACTCTTGCTGGTACAATATTGATTGAAAGAGAGTGGTTGTTAGTTATATCT
GAAGGTCAACCCCCAGAATTTCTTACAAAGATGAATTCAGTAACGAGACGCATTGATCT
AACCTGCAAGCTTCTTGCTCCTGTTGTTACTGGGTTCATAATAAGCTTTGTATCACTGAA
AGCATCTGCTATAACTTTAGCACTATGGAATACTGTGTCTGTTTGGGTGGAGTATTGGCT
TTTCACATCTGTATATAAAGGGATTCCAGCTTTGGGTCAAAGTAGCCAGAGGAGAATGG
CAAGACTTTTAGAAAGTGATCAAGAAAGGAGCAATCCAACATTGGAGGGAGATAGATT
GCTTCCTGTTACTGATGGTAGCTCAGAATTGGTAGATAGAAAATGCAGTAAAAAACTTT
ATGAGAAGATTTCAGAGATTCCTTATATTGCTGCATGGAGAGTATATTTACAGCAAGAA
GTTGTCCTTCCTGGACTAGCTCTGGCTTTGCTATTTTTTACTGTACTCAGCTTTGGAACTT
TGATGACAGCCACCTTAGAATGGGAAGGGATACCTGCATACGTTATTGGATTAGCAAGA
GGAATAAGCGCTCTGATTGGAATAGCTGCAACAATTGTATATCCTCTGCTACAGTCTCAT
ATATCAAGTATTAGAACTGGACTGTGGTCTATATGGTCTCAGTGGACTTGCCTTCTTCCA
TGTATAGCTGCAATTTGGATCCAAAATGGCTTTCTATCATCATATATTCTGATGGGAAGT
GTAGCCATATCTCGGCTTGGATTGTGGATGTTTGATTTGTCTGTGCTTCAACAAATGCAG
GATCTTGTTCCTGAATCTGATCGTTTGATTGTTGGAGGTGTTCAGAACTCACTTCAGTCA
TTGATGGACTTATTGGCCTATGTTATGGGAATCATATTATCTGATCCAGGGGACTTCTGG
AAGTTGACTTTGTTATCATTTCTAGCAGTCACATTAGCTGCATTCCTCTACTGCATCCATG
TATACCGTGTGCGAAAGCACATCTTTCACTTCGATCGTGTTATGTGGAGCAAATGTTTTC
TAGGAGCATCTTAA

Coding sequence (CDS) of *GmIREG3* is 1509 bp which was cloned with the probe sequence using HX3 line of *Glycine max* and sequenced by Sangon Biotech.

1.2 Promoter sequencing information of *GmIREG3*

GACTTAGACACGATGCAGATTGAAGAATTACAACATTCTCTTGAGGCACATGAGATGAC
AATCAATAAGTGAAGATCCCATCAAGAACAAGTACTTCATGTACATTCCAATTACAAAG
GGAAAGAACCATGGAAGGGAAACAAGTCAAACACCACCACAAAGAACCAGGAACAAG
TGATAGTTGAAGGTAGTGAATCCTCCAAAGGAAAAACAACATGCCAAACTTCTCAGAAA
TCAAACTGCAACAACAACAAGGAGTGGAAGCTCGACAAAAGAAAGGTGAAATGCTACA
ACTGTCAAAAACTTGGTCATTATGCAAAAGAATTTTGGCAAGGAGAAGGTGCCAAGAAC
AAACTCAATAATCATGCAAATTTGGCTCAAGATGAAGGCTCAGATTCTGAACTTGTATT
GCTTATGGCTACCATTAGCAGTGAATCATCAAGCGACTTCTCATGGTACTTAGATTCTGG
TTGCTCCACTCATATGACAAGGAAGAGGGATTGGTTCATCAATCTAAATAAATCATCCA
AGAGCTGAGTTTGCTTTGCAGATGATAGTACATTGATTGCTGAAGGTATTGAAAAAGTA

GCATTCAGAAGAAGAATGGAAAAGACACTGTCATTGAGGAGGTGTTGTTTGTACCTGGC
ATGAAGACTAATCTGTTAAGCCTAGGCCAACTACTTGAAAAGGGGTTTGTTATGAGGAT
GAATGACAATTGTCTCAAAGTGTTTGATAAAGTCCAAAAACTTGTGATTAAGACAGACT
TGTCTCAAAACAAGACCTTTAGGATTGGGATGAATATTCTGAAGCATAAGTGTCTTGCA
ACCTCAGAAAACAAGATGGAGTGGTTATGGCATCATAGATTTGGTCATCTCAACTTCAA
GGACCTATATCTTCTTACCTGACACAAAATGGTTGAAGGACTACTTCAGGTCTTAGTCCC
ACAGAGGTGTGCAAAGAGTGTATTGAATGCAAGCAAGCCAAAAGCATCTTCAACAAGT
ATATTCTCACAAATAATAATTAATTATCATGAATTGCATCTCATAGTAAATATGATACCG
GCCATTTCTCTCTCTTTTTCTCTGTATTTTTTAAATTTATAGTAGTGATATATAAATATCA
CTATTTAATAAACACAATCAATAACACTCATTCTTTTGTCTTTCTATCACATTATAATATT
TATTATGCTTATATTTCTTTTTTTCCCTCTCTTTTTAGGTGTTAAAGAGGACATGAGTGTT
CATTGATATATATTTTTCTAAATTTTATTATCAATGTAGAGACTTTTTTTAGGATATCCAT
GTAAAAACTTTATTAACTGGGTGATACTTGTGTAAGAATTCTATGAATTTTGAAATATTT
AATAATTAAATATATTTACATTATTGTCTCATTTTAATTTTCATTTTTATTGATAATAAGA
TTGTTCACTTTTATAATAATTATTTTAAAAAATTAGGATTAATTTTGATGGATTAATAAG
ATAAAAAAATCTATTATATCCATATTAAAATTAATTAAATTCATATCCTTGTTGCCGGTA
AACTTTCTAGGCTCATCTTATATATAAAAAAAAATGTACAAACAACAAATCATGAACTAT
TGATATTCAATAGATTTTCATTTAAAAATTGTACCGCGACGTGATGTTTGTGGAACGATC
CATGATCTTTTGTGCATTTCTTGTTAGCATTACTCAGTTATAACTTGGTTAGGTAACACAA
CTTAGATTCCAGGTGCTATTCAACGAAAGTATATTATTCTATAGCTTTGATAAGAGGAAA
ACTGTCTGTTTCGGCCTCCAATTATTATTATTTTATTTGTTTAATTTGTTTGTATCATTGAC
TCATCATCGTATATATGGACAAAGGACAGAAACTGCATGTTGCTTGCTTTAGATAATCTT
TGTAAAACTAATTAATGGTCAAAAGGAGTGAGTGAGGGGAAGAATCTGAT

Promoter sequence of *GmIREG3* is 1958 bp which was cloned with the probe sequence using HX3 line of *Glycine max* and sequenced by Sangon Biotech.

2 Supplementary Figures and Tables

2.1 Supplementary Figures

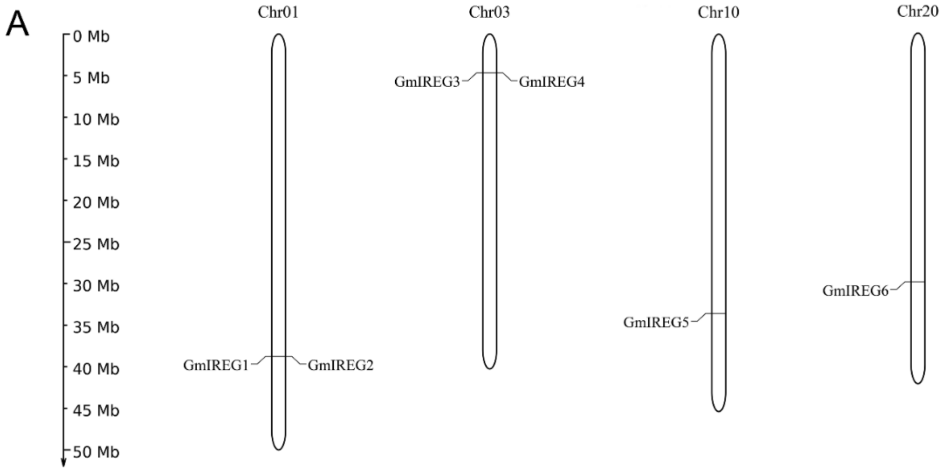

B

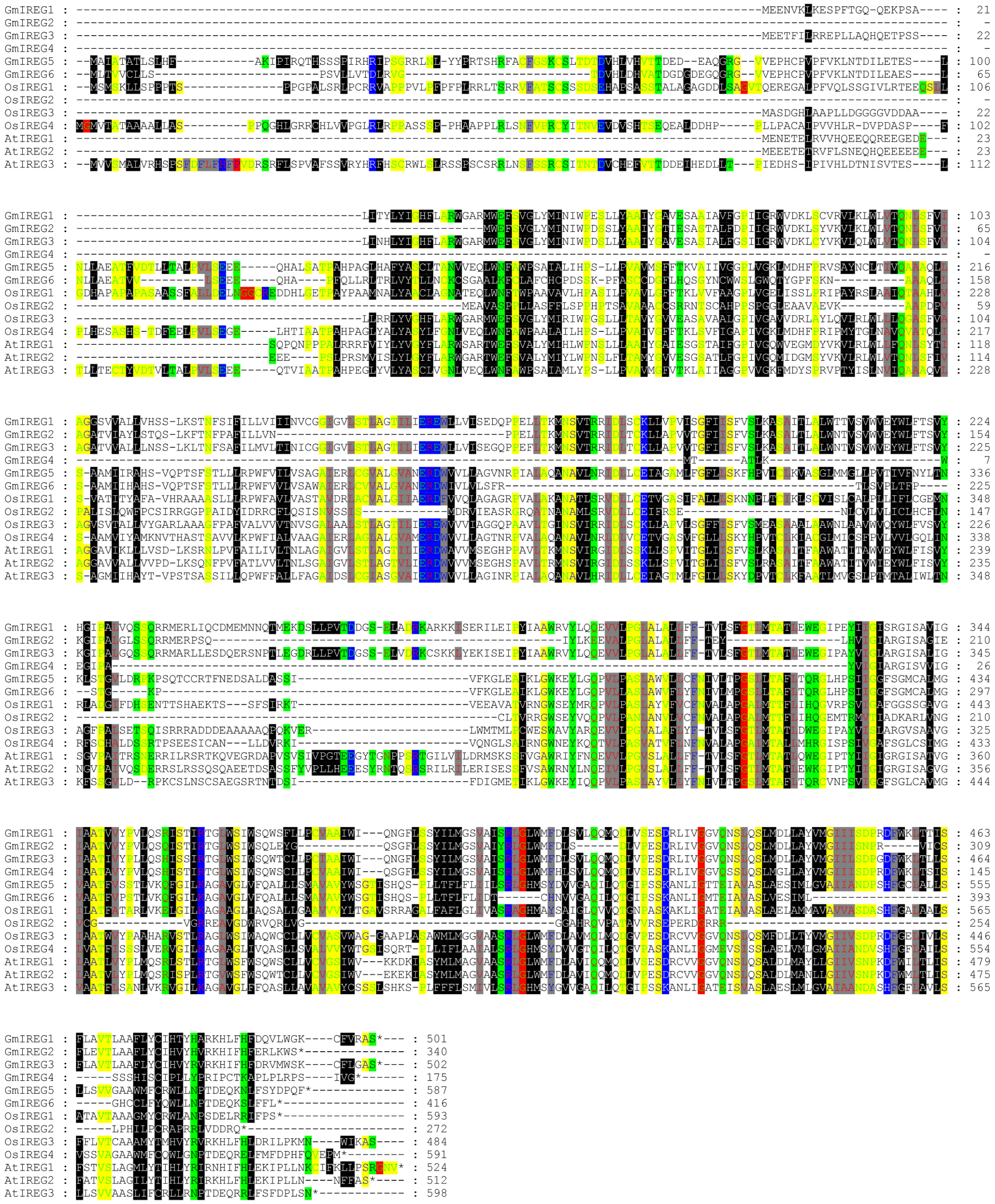

Supplementary Figure S1. Chromosomal positions and multiple alignment of soybean IREG family genes. (A) Positions of IREG family genes on soybean chromosomes. (B) Multiple alignment was performed with Clustal W and the residues were colored using Genedoc software.

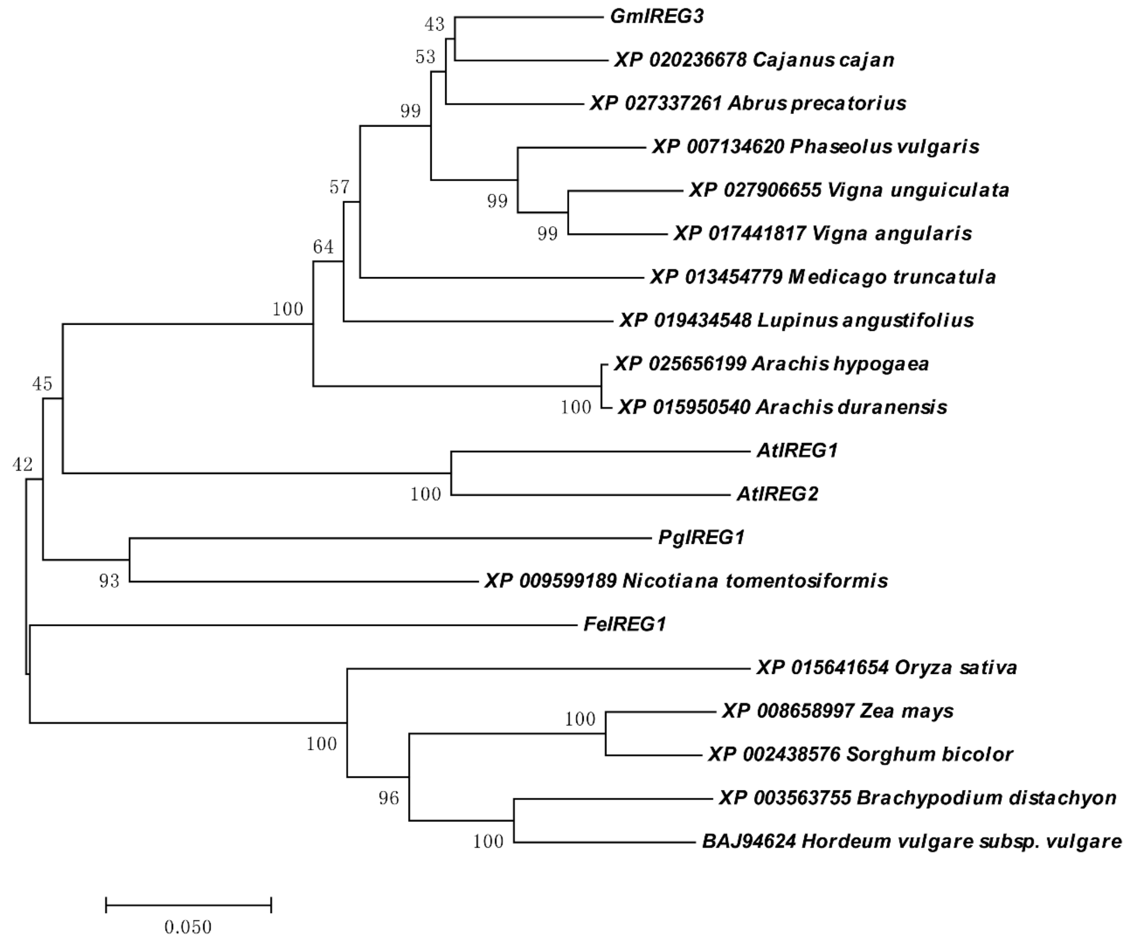

Supplementary Figure S2. Phylogenetic relationship of *GmIREG3* and the other IREG family genes from different species. The phylogenetic tree was constructed using the neighbour-joining method with 1,000 bootstrap replicates by MEGA 7.0 software. The accession numbers for IREG family genes from different species are shown with accession numbers and species, and other accession numbers are as follows: *AtIREG1* (At2g38460), *AtIREG2* (At5g03570) and *PgIREG1* (CCM80483.1). The amino acid sequence of *FeIREG1* can be found in Yokosho et al. (2016).

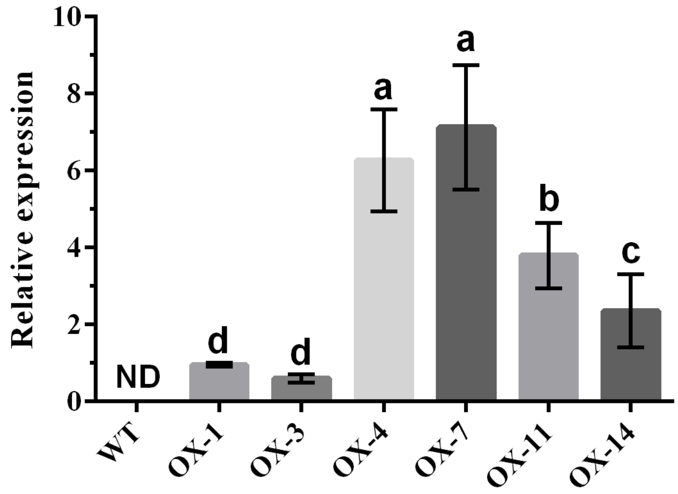

Supplementary Figure S3. *GmIREG3* overexpression in Arabidopsis. qRT-PCR determination of *GmIREG3*-overexpressing Arabidopsis lines. Data are means ± SD of three biological replicates. Mean differences are significant at the $P_{0.05}$ level between the bars with different letters and not significant at the $P_{0.05}$ level with the same letters.

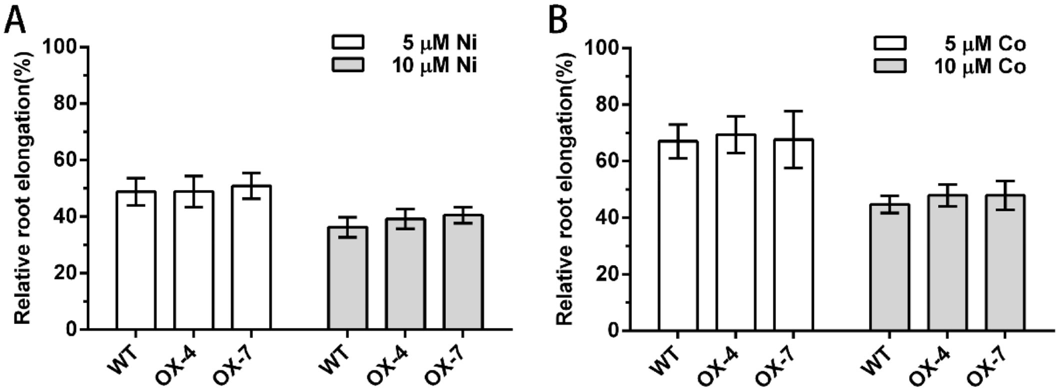

Supplementary Figure S4. Ni and Co tolerance of Arabidopsis over-expressing *GmIREG3*. Seedlings were grown on a plate containing 1 mM $CaCl_2$ (pH 5.0) in the presence of $NiCl_2$ (0, 5, and 10 μM) (A), or $CoCl_2$ (0, 5 and 10 μM) (B). And then root elongation was measured at 3 days after the exposure with a ruler. Root elongations relative to Ni and Co are shown. Data are means ± SD (n = 7).

2.2 Supplementary Tables

Table S1. Primers used for PCR and real-time PCR.

Primer name	Primer sequence (5'-3')
qGmIREG1F	TGCAACAGTTGTATACCCTGTG
qGmIREG1R	GATAAGTCAAACATCCACAATCC
qGmIREG2F	CTCCACTGCACTGTTTGATCC
qGmIREG2R	GGGGGTTTACCAGCAATATGA
qGmIREG3F	GCCTCTTCTTGCCCAACATC
qGmIREG3R	GCAAAACCTTCACATAACACAAC
qGmIREG4F	TGCAACAGCTGTATATCCTGTG
qGmIREG4R	TTGAAGCACAGACAAATGAAAC
qGmIREG5F	TCGTCAAACGCATTCATCTT
qGmIREG5R	CTCATCCTCATCCGTGGTAA
qGmIREG6F	TTGTGCCTTCAACTCTGGTC
qGmIREG6R	TTGCTTTGGATGATGGGATT
GmActin6F	GCACCACCGGAGAGAAAATA
GmActin6R	GTGCACAATTGATGGACCAG
AtActin2F	GGTATTGTGCTGGATTCTGG
AtActin2R	CGCTCTGCTGTTGTGGTGA
Clone-GmIREG3F	GTCAAAAGGAGTGAGTGAGGG
Clone-GmIREG3R	GATCCAAGAGCCACTATGTCA
pTF101-GmIREG3F	gagaacacgggggactctagaATGGAAGAAACCTTCATATTGAGGA
pTF101-GmIREG3R	cgatcggggaaattcgagctcTTAAGATGCTCCTAGAAAACATTTGC
Promoter-GmIREG3F	TTGTGATTGAAGAAAGAAGGTGGTG
Promoter-GmIREG3R	TGATGTTGGGCAAGAAGAGGC
P3301-proIREG3F	tatgaccatgattacgaattcTTAGACACGATGCAGATTGAAGAAT
P3301-proIREG3R	ttaccctcagatctaccatggATCAGATTCTTCCCCTCACTCACT
p1302-GmIREG3F	acgggggactcttgaccatggCTATGGAAGAAACCTTCATATTGAG
p1302-GmIREG3R	aagttcttctcctttactagtAGATGCTCCTAGAAAACATTTGC
